# New Mode of Opening an Ovarian Cyst

**Published:** 1855-04

**Authors:** 


*New Mode of Opening an Ovarian Cyst.*—Dr. Sims, of this city, (New York,) has recently performed this operation in a mode which is new and which seems to possess several excellencies. A trocar, fifteen inches in length, curved so as to be the arc of a circle of four and a half inches radius, was, with its canula, inserted at the usual place of tapping in abdominal dropsy. After a portion of the fluid was drawn off, the point of the trocar was drawn within the canula, which, after several attempts, was finally carried to the cul-de-sac, between the uterus and rectum; and when felt there by the finger in the vagina, the trocar was again produced, the sac and vagina perforated, and the extremity of the canula brought out between the labia majora; thus, in fact, transfixing the patient. A self-retaining catheter was then attached to the canula, and drawn into the ovarian sac, where, being separated from the canula, it was secured within the sac. The canula was then withdrawn, and the external opening closed. In this way the contents of the cyst were allowed to drain entirely away, and it is hoped that obliteration of the sac and the cure of the patient will be the result. At our present writing, nearly three weeks after the operation, she is going on well.—*American Medical Monthly.*

